# Physical and Mental Health Impacts of Household Gardens in an Urban Slum in Lima, Peru

**DOI:** 10.3390/ijerph15081751

**Published:** 2018-08-15

**Authors:** Abigail Korn, Susan M. Bolton, Benjamin Spencer, Jorge A. Alarcon, Leann Andrews, Joachim G. Voss

**Affiliations:** 1 School of Public Health, University of Washington, Box 352100, Seattle, WA 98195, USA; akorn@uw.edu; 2 School of Environmental and Forest Sciences, University of Washington, Box 352100, Seattle, WA 98195, USA; sbolton@uw.edu; 3 Department of Landscape Architecture, University of Washington, Box 355734, Seattle, WA 98195, USA; bspen@uw.edu; 4 TRACTION, Parque Leon Garcia 177, Pueblo Libre 15084, Lima, Peru; cocoa84@uw.edu; 5 College of Built Environment, University of Washington, Seattle, WA 98105, USA; andrewsl@uw.edu; 6 Sarah Cole Hirsh Institute for Evidence-Based Practice, Frances Payne Bolton School of Nursing, Case Western Reserve University, 2120 Cornell Road, Cleveland, OH 44106, USA

**Keywords:** green space, mental health, Peru, quality of life, urban slum, social capital

## Abstract

Rural poverty and lack of access to education has led to urban migration and fed the constant growth of urban slums in Lima, Peru. Inhabitants of these informal settlements lack land rights and access to a public water supply, resulting in poor sanitation, an inability to grow food, and suboptimal health outcomes. A repeated measures longitudinal pilot study utilizing participatory design methods was conducted in Lima between September 2013 and September 2014 to determine the feasibility of implementing household gardens and the subsequent impact of increased green space on well-being. Anthropometric data and a composite of five validated mental health surveys were collected at the baseline, 6-months, and 12-months after garden construction. Significant increases from the baseline in all domains of quality of life, including: physical (*p* < 0.01), psychological (*p* = 0.05), social (*p* = 0.02), environmental (*p* = 0.02), and overall social capital (*p* < 0.01) were identified 12 months after garden construction. Life-threatening experiences decreased significantly compared to the baseline (*p* = 0.02). There were no significant changes in parent or partner empathy (*p* = 0.21), BMI (*p* = 0.95), waist circumference (*p* = 0.18), or blood pressure (*p* = 0.66) at 6 or 12 months. Improved access to green space in the form of a household garden can significantly improve mental health in an urban slum setting.

## 1. Introduction

### 1.1. Impacts of Gardening on Health in Urban Slums

The impacts of gardening and access to green space on various aspects of physical and mental health are well documented. Participation in gardening activities can increase the availability of fresh fruits and vegetables in “food deserts” within low-income communities [1,2]; improve food security [3]; have protective effects against the onset of chronic, non-communicable diseases (NCDs) including cardiovascular disease, diabetes, and cancer [4,5]; reduce stress [6]; facilitate social inclusion and community connection [7]; and improve the overall air quality [8]. Green space in the form of urban gardens has been shown to improve access to nutritious food [9,10] and, if the gardens are large enough, to shield slum dwellers from volatile food prices [3]. Barnidge et al. (2013) conducted a study in Missouri showing that 95% of gardeners surveyed were twice as likely to meet the daily recommendations of fruit and vegetables as compared to participants who did not garden [9]. A population-based, cross-sectional study in Utah showed that persons participating in community gardening activities had significantly lower body mass indexes (BMIs) than their non-gardening neighbors and siblings. The BMIs of female gardeners were 1.48 times lower than their neighbors who did not garden and 1.88 times lower than their sisters on average; male gardeners had BMIs 2.52 times lower than their neighbors and 1.33 times lower than their brothers [11]. Weltin (2013) conducted a 6-month intervention study in Iowa with immigrants from the Marshall Islands suggesting that type 2 diabetes could be combatted via community gardening. Following the intervention, glycated hemoglobin levels (HgBA1C) among diabetic participants had dropped from 8.2 to 6.6%, while the diabetic control group who did not garden experienced worsening HgBA1C levels from 9.3 to 9.9% [12]. Previous investigators have found that in Peru, a low socioeconomic status is strongly associated with higher burden of NCDs such as type 2 diabetes and cardiovascular diseases [13,14], and it is well documented in the health literature that reductions in BMI and increased consumption of fruits and vegetables can reduce the risk for the development of these chronic conditions. These findings suggest that increased consumption of fruits and vegetables via household and community gardening mechanisms could have implications for the prevention and management of NCDs in Peru. As the burden of NCDs increases in low- and middle-income countries (LMICs), more community-based interventions to better prevent the onset of NCDs in these settings are needed [15]. Slum residents, most of whom have weakened familial connections and may come from different cultural backgrounds, rely on social cohesion to generate social support and to catalyze collective action within their neighborhood, especially in the absence of government support [16].

Previous reviews have documented that access to green spaces improves the objective and self-reported health outcomes through mechanisms including significantly reducing stress and promoting social cohesion in neighborhoods [6]. Although urban centers provide a dense network of resources, there are also unique urban stressors like noise pollution and fear of crime. Researchers suggest that people’s affinity for green space within cities stems from its restorative function which alleviates and reduces stress [17]. A recent review highlights the association between individual mental health outcomes and the perceived quality, not just quantity, of green space [6,18]. Whatley, Fortune, and Williams (2015) found that gardens facilitate social connection and participation and promote social learning by creating a flexible environment that supports individuals with diverse cultural backgrounds and life experiences [7]. Within these gardening communities, members feel safe, accepted, valued, and a sense of belonging and contribution to a broader purpose [7].

Finally, from an environmental perspective, trees, shrubs, and other plants filter out pollutants including ozone, particulate matter, and nitrogen dioxide, therefore, improving air quality [8]. They also provide shade and protect people from heat and sun-related illnesses, including skin cancer [19,20].

### 1.2. Urban Slums in a Peruvian Context

Of Lima’s 9.7 million inhabitants, an estimated 85% live in informal settlements [21]. Informal settlements “(i) were built in violation of express laws, (ii) did not comply with requirements for access to land, (iii) were originally formal but became informal, or (iv) were built by the government without complying with legal requirements” [22]. Many residents of these settlements live in poverty and they qualify as slum households; defined by the United Nations as “a group of individuals living under the same roof in an urban area who lack one or more of the following: (i) durable housing of a permanent nature that protects against extreme climate conditions; (ii) sufficient living space which means not more than three people sharing the same room; (iii) easy access to safe water in sufficient amounts at an affordable price; (iv) access to adequate sanitation in the form of a private or public toilet shared by a reasonable number of people; and (v) security of tenure that prevents forced evictions” [23]. Slums in Peru often begin as “invasions,” with a group of families constructing makeshift houses on vacant land. To apply for the land title, neighborhoods must work together to bring the area into compliance with local regulations such as land stabilization and the mapping of formal land parcels. With formalization and recognition from the city comes access to public water and sanitation, road construction, educational, and health services. People living in slums face complex intertwined issues including food and water insecurity, geographic and political marginalization, and poorer health outcomes [24].

Coastal Peru receives less than one inch of rainfall per year and is particularly vulnerable to this explosive urbanization. Water for Lima’s 9.7 million people comes from rivers that are fed by Andean glaciers, which are melting at an unprecedented rate [25]. The combination of increasing water insecurity, changing ocean temperatures, and rapid urbanization makes healthy urban ecosystems and increased green space progressively more important. Furthermore, green space is critical to preventing erosion, landslides, and soil degradation. However, in the arid Lima area, green space is limited and concentrated in affluent areas. Within slum neighborhoods, access to green spaces is extremely limited, placing residents and their families at greater risk for mental illness, social isolation, increased stress, and reduced quality of life [26]. Urban gardens have gained popularity for their potential to improve physical, mental, and environmental health in low-income settings [6,17]. Although many studies have been conducted in poor neighborhoods in high-income countries, little research has been done on green space interventions in low- and middle-income countries (LMICs) like Peru, despite evidence of multiple positive impacts of such interventions on physical health and mental well-being detailed previously [27]. Alto Zapallal is in the northern cone of Lima and home to approximately 30,000 residents. One of the newest settlements is Eliseo Collazos, where the first houses appeared between 2006–2007. Eliseo Collazos was built around the slopes of a former clay mine and is a growing informal urban settlement of about 90 families. Residents have purchased and registered their lots but are in the process of gaining property titles. Most families have built prefabricated houses on unstable hills. The majority of homes have electricity, but the neighborhood is not connected to municipal water or sanitation services. Historically, water was delivered through a central hose or brought in by trucks and sold by a private water company. Sanitation is limited to outhouses and makeshift showers.

### 1.3. Project Stakeholders

To gain the trust of individuals in Eliseo Collazos, this project engaged the community as well as academic and local partners through the Informal Urban Communities Initiative (IUCI). IUCI is a partnership between the University of Washington, the Fundación San Marcos in Peru, and various neighborhoods in Alto Zapallal. Previous projects have focused on other communities within Alto Zapallal. The purpose of IUCI is to design, implement, and assess community-based interventions to improve human and environmental health. IUCI has used participatory design methods for landscape, engineering, and health projects in the northern cone of Lima since 2010 [28].

### 1.4. Objectives

This 1-year pilot study aimed to investigate the effects of individual household gardens on the health of urban slum dwellers in Lima, Peru via the following three objectives:

1. To identify any changes in physical health status at 6 and 12 months after the introduction of individual gardens as measured by increased or decreased BMI, fasting blood glucose, blood pressure, and waist circumference.

2. To assess mental health changes at 6 and 12 months after the introduction of household gardens as measured by validated indicators including perceived stress, empathy towards their partners and their children, threatening life experiences, quality of life, and social capital.

3. To determine if any baseline demographic variable predicted the construction of a garden by participants.

## 2. Materials and Methods

### 2.1. Study Design

A longitudinal study with repeated measures taken at the baseline, 6 months, and 12 months post garden construction was conducted. Information was collected in questionnaire form via one on one interviews with each participant. Participants could opt to verbally respond to questionnaire items asked by the interviewer or to read and complete the questionnaire on their own.

### 2.2. Measurements

Demographic information collected included age, gender, marital status, education, monthly household income, number of adults in household, number of minors in household, years lived in the community, language(s) spoken, province of origin, previous residence, reason for moving, whether participant was currently ill, alcohol and smoking status, and self-reported physical activity, and problems with mouth and teeth.

The physiological indicators collected included basic biometric measurements of height, weight, waist circumference, resting blood pressure, and fasting blood glucose using standardized procedures. The same scales, blood pressure cuff, and blood glucose meter were used for all participants at all time-points.

### 2.3. Mental Health Instruments

Mental health dimensions were assessed using the Spanish translation of several tools: the World Health Organization Quality of Life-Brief Version (WHOQOL-BREF); the Perceived Stress Scale (PSS); the Life-Threatening Experiences Scale (LTE-Q); the Social Capital Scale (SCS); and the Parent/Partner Empathy Scale (PPES). The combined questionnaire was validated in the community and adapted for local use. The WHOQOL-BREF has four quality of life subscales: quality of life in the physical domain, the psychological domain, the social domain, and the environmental domain. This is the 26-item version, scored on a 5-point scale, with higher scores indicating a higher quality of life [29,30]. The PSS measures the degree to which the interviewee considers her/his situation stressful. The instrument has 14 items, and uses a 5-point response scale, with higher scores indicating higher levels of perceived stress [31]. The LTE-Q asks whether 12 yes/no life events have occurred in the past three months. Higher scores indicate more recent life-threatening experiences [32,33]. The SCS quantifies social capital through eight subscales: participation in the local community, social agency, feelings of trust and safety, neighborhood connections, friends and family connections, tolerance of diversity, the value of life, and workplace connections. Example questions include, “How comfortable do you feel walking alone at night in your neighborhood?” and “In the last week, have you visited a neighbor?” Workplace connections items were excluded in anticipation of the high rate of informal work in the slum community, leaving a 36-item questionnaire, which uses a 4 point response scale. Higher scores indicate higher levels of social capital [34]. The PPES uses a parent subscale and a partner subscale. There are 40 items with a 5-point response scale. Subjects were asked to respond to how well each item describes them. For example, “My partner says I don’t understand what they feel,” or “When my child is bothered, it is hard to tell if s/he is sad or just nervous.” Higher scores indicate higher levels of empathy [35].

### 2.4. Recruitment Procedures

The study was announced through community meetings and posted flyers in June 2013. All community members were invited to a presentation that introduced the details of the study design. To participate in the project, participants had to commit to attending workshops on four consecutive Sundays. In a community where most residents work six days a week, this constituted a huge time commitment on the participants’ part. The individual garden design and participatory workshops that led up to the garden planning and construction are described in detail elsewhere [36]. Briefly, participants attended four Sunday workshops of 2–3 h during July 2013 to envision what they wanted their community to look like in the future, to design a model of their personal garden with scaled models, and to learn about construction and cultivation methods. Following the presentation, researchers were available to answer the community members’ questions. Informed consent was obtained from residents that were interested in participating. The inclusion criteria included attending all workshops, age 18 years or older, ability to give informed consent, and residence in Eliseo Collazos. One participant attended workshops and constructed a garden but was unable to complete the Spanish questionnaire because she only spoke an indigenous language. A total of 44 eligible residents out of 90 households signed informed consent to participate. Each participant used the available space around their house and the available $150 budget for the construction of the garden. Each participant had the option to spend the available funds on soil, fencing material, seeds, plants, and netting as desired. Not every participant chose, in the beginning, to fence their garden, however, at the end of the project every participant had fenced their gardens to protect them from feral dogs, cats, and chicken and to provide some privacy. Each garden was designed based on the functionality that each owner hoped to gain out of the garden, including to grow food, flowers, trees, or feed for small animals.

### 2.5. Ethics Approval

Previous experiences including community meetings in 2011 and projects at the local school facilitated trust and support for the project. This community-driven project identified a lack of green space as one of their top priorities through a participatory impact assessment conducted in 2011. In 2013, the Institutional Review Boards of the University of Washington (IRB Protocol #44864), the University of San Marcos, and the community members approved the study. 

### 2.6. Statistical Analysis

A paired t-test was utilized to show changes in mean scores on all physiologic and mental health indicators from the baseline to 6-months and the baseline to 12-months post garden construction. Baseline demographics of participants who completed workshops and built a garden were compared with the demographics of participants who did not build a garden. The mean comparisons were not adjusted for any demographic variables due to the small sample size.

## 3. Results

Baseline data was collected for 44 adult community members. Of these participants, 29 completed all the workshops and constructed a garden on the perimeter of their house (Appendix A). Those who did not build a garden were younger, more likely to be male, single, and had a lower monthly income when compared to the residents who did construct a garden (Table 1). To determine whether or not there were underlying differences between people who built a garden and people who did not ultimately construct a garden, we present the stratified data.

Changes in the biometry from the baseline to 6-months and the baseline to 12-months among those who constructed a garden are shown in Table 2. There was no significant change in BMI, waist circumference, or blood pressure at either follow-up. The mean BMI fell at the high end of the normal range at all-time points. Waist circumference varied among subjects, but the mean at baseline and 12-months was slightly higher than the recommended 88.9 cm. During all three measurements, the mean fasting blood glucose fell within the normal range (79.2–110 mg/dL). At 6-months, we detected a significant increase in mean fasting blood glucose, but at 12-months, the change in mean fasting blood glucose was non-significant. The mean blood pressure fell below the recommended 120/80.

Results from the paired t-test for mental health variables are shown in Table 3. There was an increase in all domains of quality of life at 6-months that was not statistically significant. However, this increasing trend in the quality of life improvement was significant in all domains at 12-months. Reports of life-threatening experiences decreased significantly on both the 12-item scale from the baseline to 12-months. Perceived stress scores increased significantly at 6- and 12-months (*p* < 0.01).

For the Parent Partner Empathy Scale, participants who self-identified as parents or partners changed over time. The PPES analysis only includes subjects on whom we have complete follow-up information. Four subjects were excluded due to incomplete follow-ups. Three surveys were incomplete but were included in the analysis with missing items omitted.

Mean social capital scale scores increased slightly at 6-months and significantly at 12-months (*p* < 0.01). We also saw significant increases in three subscales at 12-months: the feelings of trust and safety (*p* = 0.01), the value of life (*p* = 0.01), and the family and friends’ connections (*p* = 0.04).

## 4. Discussion

We found that, at 12-months post-construction, the gardens were associated with significantly better mental health scores as measured by improved quality of life, reduced threatening experiences, and increased social capital. The improvement in mental health is consistent with other studies and demonstrates the benefit of this environment [6,27]. Previous investigators have proposed a relationship between decreases in the number of life-threatening events and the presence of individual gardens [37]. They indicated that the gardens promote more socializing and public presence in the neighborhood which reduces stress levels and violence potential among community members [16,38].

The quality of life and social capital scales were the most sensitive to change, indicating that the environmental change had the biggest impact on the individual and the community. Understanding how environmental changes influence social networks to improve wellbeing and accelerate development will be critical as more people move into urban centers and informal settlements. This participatory design of household green space intervention methodology was feasible and highly regarded amongst the participants. It allowed them to make their own choices regarding garden placement, design, and content. These results also point to the synergy between the co-benefits of improving environmental health and human health.

Perceived stress increased significantly, more at 6-months than at 12-months. Based on the direct observation, this result may reflect community members’ perception of increased gang presence. The peak in perceived stress at 6-months also may reflect the difficulties of the hot, dry season and the impending start of school and related expenses.

Looking at physical health, we found that BMI in this community was lower than in a previous study in the Alto Zapallal area. A total of 30% of our participants were overweight or obese at baseline as compared to the 53% in the previous Alto Zapallal study. In the same study, 15% of participants had high blood pressure whereas only 2% of Eliseo Collazos subjects had high blood pressure [13]. However, as a newer settlement, there may be important differences in variables like distance to catching public transportation, the absence of a drivable street, a mainly younger age and better general health in Eliseo Collazos compared to the larger Alto Zapallal area that has been established decades ago. For example, it takes 30–45 min to walk to the bus station to go to central Lima.

Fasting blood glucose levels suggest a low incidence of type 2 diabetes in Eliseo Collazos. There was a statistically significant but not a clinically meaningful increase in fasting blood glucose at 6-months, but not at 12-months, which may be associated with varying seasonal eating patterns. Residents report regular exercise from climbing the sandy hills in the neighborhood that cars and buses cannot navigate. To better assess the impact of gardens on physical health, future projects should also collect data on baseline nutrition and describe food intake at various time points. Furthermore, families did not exclusively use their gardens for food, differing from much of the literature on urban gardens. The expected changes could, therefore, be mediated through mental health, and we were not powered to observe a significant mediated change in the basic biometry at 12 months. In this participatory design where subjects chose their own plants, it would be impossible to conduct an a priori sample size calculation. The garden output and the nutritional value will be presented in a later manuscript.

Underlying differences between participants who implemented the garden and those who did not suggest that this methodology may not benefit all community members, particularly single adults with lower monthly incomes. The proportion of bilingual residents was equal in the group that constructed a garden and the group that did not, so language does not appear to be a limiting factor in the population that benefited from the project. We saw in this community that single adults had less flexibility in their daily schedules due to their focus to generate sufficient income. Following the implementation of workshops and construction of study gardens, other community members not in the study constructed new gardens in front of their homes. Future studies should record characteristics of all neighborhood households to better document community-level changes.

### Limitations

Although the sample size for this study was small, 30% of all households in the neighborhood constructed a garden, representing a significant proportion of the community evaluated. Limited study resources did not allow for follow-up on participants who did not construct a garden to evaluate their reasons. Most likely, these heads of households were not able to dedicate the significant amount of necessary time required in the initial workshops. However, significant associations were still detected, suggesting that the garden project made a sizeable impact on the well-being of residents.

The scales employed were not all developed for use in our study setting. Some were validated translations, while others were geared towards populations of a higher socioeconomic and education level. For example, one question asks how many phone conversations with friends the participant has had in the past week. In our population, not all residents own cell phones, so we coded this answer as zero when, in reality, the correct answer is “not applicable.” This could be remedied by creating a data analysis plan that includes a “not applicable” (N/A) option and details appropriate statistical tests.

The participatory design appeared to be a critical aspect of the study. In marginalized populations, this methodology empowers community members. Although requiring that families attend workshops created a bottleneck for participation, utilization of this methodology allowed families to customize the design and content of their gardens which may increase the long-term sustainability of the garden projects in Eliseo Collazos.

Finally, a particularly important variable for garden interventions in arid regions is access to water. Studies conducted in desert areas should include metrics on how much and what types of water are used for gardening to ensure the sustainability of the green spaces. We did not capture the use of water in metric quantities but in a qualitative format (used a little, moderate, or a lot of water). This did not allow us to quantify the absolute amount of weekly water usage but rather their perception of how much water they used based on their subjective assessment. This data will be presented in the following manuscript as well.

## 5. Conclusions

Our results highlight the interplay between the individual, family, neighborhood, and societal levels of influence on physical and mental well-being in a population of marginalized urban slum dwellers in Lima, Peru. Participatory green space interventions are feasible and can have significant health benefits in these low-resource and low-income communities. Future research could examine green space in a cohort study to better examine the changes in well-being that occur over time or throughout a community, regardless of whether the resident has a garden. Further investigation into dietary and physical activity patterns before, during, and after a garden intervention would provide a more nuanced understanding of the health impact.

## Figures and Tables

**Table 1:** The baseline demographics of study participants.

Characteristic	Did not Construct a Garden *N* = 15		Constructed a Garden *N* = 29	
	*n*	%	*n*	%
Age in years (mean, range) *	31.9	19–58	34.5	21–58
Education * (years) ^†^	10.6	1–22	9.3	0–20
Gender				
Male	2	6.9	2	13
Female	27	93	13	87
Language				
Spanish and Quechua	3	20.0	6	20.7
Spanish only	12	80.0	23	79.3
Marital Status				
Single	5	33.3	1	3.5
Live together	6	40.0	22	75.9
Married	2	13.3	1	3.5
Separated	2	13.3	5	17.2
Monthly income (soles) ^†^				
0–374	7	46.7	6	20.7
375–749	6	40.0	16	55.2
750–1500	1	6.7	7	24.1
Missing ^††^	1	6.7	0	0
Physical activity				
0–30 min	4	26.7	6	20.7
1–2 h	3	20.0	11	37.9
2–3 h	0	0.0	4	13.8
3+ h	7	46.7	8	27.6
Missing ^††^	1	6.7	0	0

Legend: * mean, ^†^ range; ^††^ missing value.

**Table 2:** The physical health metrics at enrollment and at six and twelve months.

Variable	Baseline *N* = 29		6-Months *N* = 28			12-Months *N* = 26		
	Mean	SD	Mean	SD	*p*	Mean	SD	*p*
BMI	24.3	5.1	24.1	5.2	0.12	24.5	5.5	0.95
WC * (cm)	89.4	13.4	87.2	8.5	0.40	92.8	13.2	0.18
FBG ^†^ (mg/dL)	89.9	8.9	94.0	**7.2**	0.04 **	91.1	8.5	0.59
Systolic	115.2	11.4	115.5	9.0	0.75	114.6	15.9	0.66
Diastolic	72.3	10.1	73.0	10.0	0.77	73.8	9.4	0.69

Legend: * Waist Circumference; † Fasting blood glucose; ** *p* < 0.05; bold means significant differences at 6 but not 12 months.

**Table 3:** The mental health metrics at enrollment and at six and twelve months.

Variable	Baseline *N* = 29		6-Months *N* = 28			12-Months *N* = 26		
	Mean	SD	Mean	SD	*p*	Mean	SD	*p*
WHOQOL-BREF *								
Physical	13.3	2.0	13.4	2.8	0.67	**15.5**	**2.4**	<0.01 **
Psychological	14.3	2.3	14.7	2.4	0.24	**15.5**	**2.4**	<0.05 **
Social	13.3	3.0	13.7	3.1	0.51	**15.1**	**3.0**	0.02 **
Environmental	11.8	1.5	12.0	1.7	0.56	**13.0**	**1.7**	0.02 **
LTE-12 ^†^	2.0	1.7	2.1	2.2	0.85	**1.5**	**1.5**	0.02 **
Perceived Stress Scale	23.8	**6.5**	**34.0**	**4.9**	<0.01 **	**33.1**	**4.1**	<0.01 **
PPES ^§^ (*n* = 19)	141.5		137.2		0.15	145.6		0.21
Parent (*n* = 24)	65.3		**61.4**		<0.01 **	63.0		0.19
Partner (*n* = 19)	65.2		65.9		0.74	68.0		0.08
Social Capital Scale	72.5	10.0	75.3	9.2	0.13	**83.6**	**10.1**	<0.01 **
Trust/Safety	18.9	3.0	19.4	1.9	0.48	**20.3**	**2.8**	0.01 **
Participation	12.4	4.3	11.3	3.7	0.17	12.5	4.1	0.88
Diversity	6.0	1.4	6.0	1.2	1.00	6.4	1.3	0.31
Neighborhood	4.5	2.0	4.9	1.9	0.38	5.2	2.1	0.16
Value of Life	11.8	1.8	11.6	2.2	0.62	**13.2**	**1.9**	0.01 **
Family/Friends	6.1	1.8	6.7	1.6	0.13	**7.1**	**1.8**	0.04 **
Social Agency	2.0	0.9	1.9	0.9	0.81	1.9	0.9	0.79

Legend: * WHOQOL-BREF: World Health Organization Quality of Life Brief Version; ^†^ LTE-12: Life-Threatening Experiences, 12-item; ^§^ PPES: Parent/Partner Empathy Scale, ** *p* < 0.05; bold means number statistically significant.

**Figure A1:**
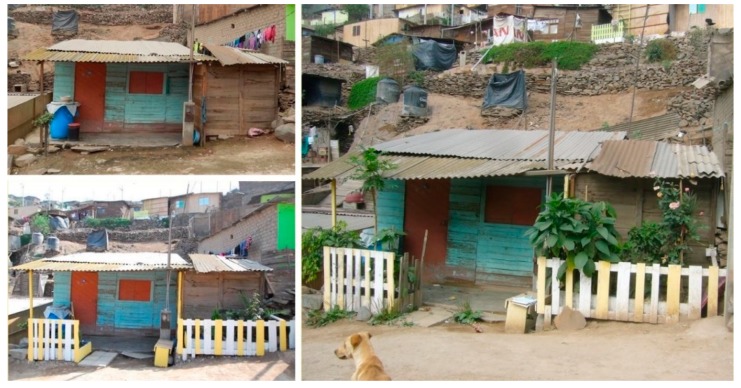
Example 1: Garden progression over a 6-months period.

**Figure A2:**
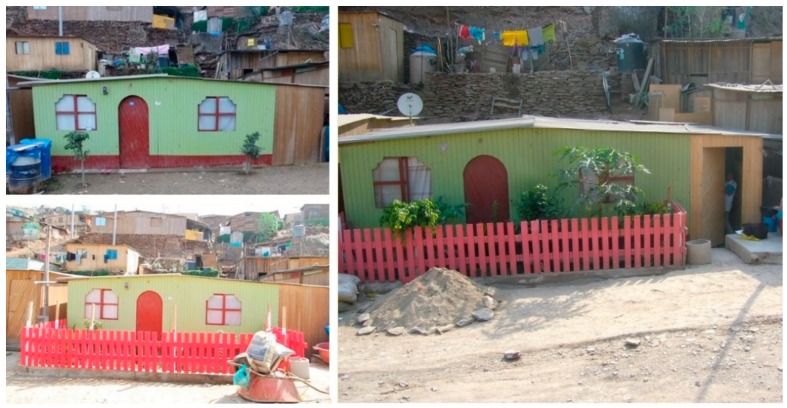
Example 2: Garden progression over a 6-months period.
